# Effectiveness of training in guideline-oriented biopsychosocial management of low-back pain in occupational health services – a cluster randomized controlled trial

**DOI:** 10.5271/sjweh.3959

**Published:** 2021-06-29

**Authors:** Katja Ryynänen, Petteri Oura, Anna-Sofia Simula, Riikka Holopainen, Maija Paukkunen, Mikko Lausmaa, Jouko Remes, Neill Booth, Antti Malmivaara, Jaro Karppinen

**Affiliations:** Medical Research Center Oulu, Oulu University Hospital and University of Oulu, Oulu, Finland; Institute of Health Sciences, Center for Life Course Health Research, Medical Faculty, University of Oulu, Finland; Department of General Medicine, the South Savo Social and Health Care Authority, Mikkeli, Finland; Faculty of Sport and Health Sciences, University of Jyväskylä, Jyväskylä, Finland; Faculty of Medical and Health Sciences, University of Linköping, Linköping, Sweden; Finnish Institute of Occupational Health, Oulu, Finland; Faculty of Social Sciences (Health Sciences), Tampere University, Tampere, Finland; Centre for Health and Social Economics, Finnish Institute for Health and Welfare, Helsinki, Finland; Rehabilitation Services of South Karelia Social and Health Care District, Lappeenranta, Finland

**Keywords:** implementation research, risk stratification, STarT Back Tool, Örebro Musculoskeletal Pain Screening Questionnaire

## Abstract

**Objective:**

This study aimed to investigate the effectiveness of brief training in the guideline-oriented biopsychosocial management of low-back pain (LBP) in occupational health services using a cluster-randomized design. A small sample of physiotherapists and physicians from the intervention units (N=12) were given three- to seven-day training focusing on the biopsychosocial management of LBP, while professionals in the control units (N=15) received no such training.

**Methods:**

Eligible patients with LBP, with or without radicular pain, aged 18–65, were invited to participate. A web-based questionnaire was sent to all recruited patients at baseline, three months and one year. The primary outcome measure was disability (Oswestry Disability Index, ODI) over one year. Between-group differences were analyzed using linear and generalized linear mixed models adjusted for baseline-response delay as well as variables showing between-group imbalance at baseline.

**Results:**

The final study sample comprised 234 and 81 patients in the intervention and control groups, respectively at baseline, and 137 and 47 patients, respectively, at one year. At baseline, the mean duration of pain was longer in the intervention group (P=0.017), and pain-related fear concerning physical activity was lower (P=0.012). We observed no significant difference between the groups’ primary outcome measure (adjusted one-year mean difference in the ODI: 2.3; 95% confidence interval -1.0–5.7; P=0.175) or most secondary outcomes.

**Conclusions:**

Brief training in guideline-oriented biopsychosocial management of LBP for occupational health professionals did not appear to be effective in reducing patients’ symptom over one-year follow-up compared to treatment as usual.

Current clinical practice fails to effectively manage low-back pain (LBP) and despite increasing healthcare resources being devoted to it, disability due to LBP has risen by over 50% since 1990 (1). The biopsycho­social model is increasingly accepted for understanding and managing pain (2). Attention should be drawn to the complex contributors to LBP such as psycho­logical, social and biophysical factors (3). For routine use with persistent LBP, interventions that consist of non-pharma­cological treatments such as exercise therapy and cognitive-behavioral therapy (CBT) should be considered (4). As best practice, a systematic review of high-quality clinical practice guidelines for musculoskeletal pain recommended the following: ensure that care is patient-centered, screen for red flags, assess the psychosocial factors, use imaging selectively, undertake a physical examination, monitor patient progress, provide education/information, address physical activity/exercise, use manual therapy only as an adjunct to other treatments, offer high-quality non-surgical care prior to surgery, and try to enable patients to remain at work (5).

A recent review showed moderate-quality evidence that biopsychosocial interventions are more effective than education/advice for reducing disability and pain in the short-, medium- and long-term in patients with LBP, and interventions with a clear focus on psychosocial factors seem the most promising (6). However, these interventions are still in early development and their implementation in clinical practice has encountered some challenges (2, 7). The results of different training interventions have varied widely and the optimal way to train healthcare professionals (HCP) and support the implementation process is not yet clear (8–11).

Early assessment and tailored interventions in primary healthcare seem to promote a more efficient approach for preventing the development of prolonged and disabling LBP (12, 13). The recent National Institute for Health and Care Excellence (NICE) guidelines also recommend risk-based stratification and targeted interventions for different risk groups (14). The STarT Back Tool (SBT) is a brief questionnaire, which has been developed to identify patients at a higher risk of developing persistent disabling LBP in order to provide treatments according to the risk group (15). The Örebro Musculoskeletal Pain Questionnaire (ÖMPSQ) has been developed to identify biopsychosocial factors that are known predictors of work disability (16) and the short version (ÖMPSQ-short) has shown to be valid in both clinical use and research interventions (17).

Previously, the effectiveness of patient education booklets (18, 19), and return-to-work interventions (20) have been evaluated among patients with LBP in Finnish occupational health services (OHS), but no studies have been conducted on the effectiveness of biopsychosocial pain management. Using a cluster randomized design, the objective of this study was to assess the effectiveness of brief training in the guideline-oriented biopsychosocial management of LBP for OHS providers who manage patients with LBP.

## Methods

### Trial design

The study design was a two-arm cluster randomized controlled trial. Units of major Finnish private and public OHS providers were invited to participate. The Ethics Committee of the University Hospital of Oulu, Finland, approved the study (79/2017, 19.9.2017), which was performed in accordance with the Helsinki Declaration.

### Participants

**Figure 1:**
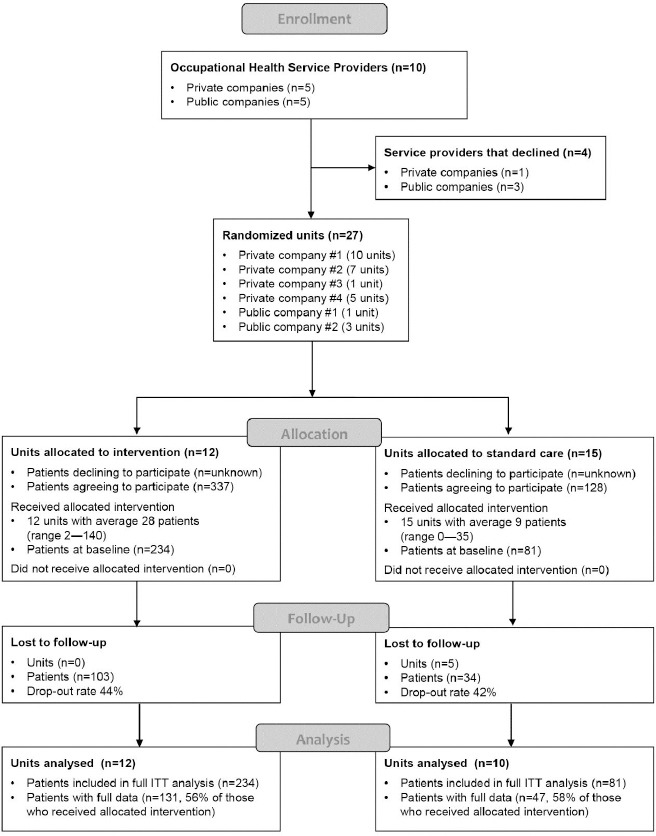
CONSORT flow chart for the study.

Figure 1 is a flowchart illustrating the progression of the study. From each unit allocated to the intervention group, at least one physician and one physiotherapist were encouraged to participate in the training. All the physiotherapists and occupational health physicians in all the study units were invited to recruit eligible patients. Inclusion criteria for patients with LBP included 18–65 years of age and the possible presence of radicular pain in addition to axial pain. Exclusion criteria included suspicion of a serious underlying cause for LBP or need for urgent care. All the participants completed written informed-consent forms, participation was voluntary, and they were not reimbursed for participating in the study. No information was collected on the patients who were invited to participate but who did not sign the consent form.

### Interventions

A small sample of physiotherapists and physicians (N=28 in the initial training and N=21 in the booster with 17 HCP participating in both sessions) from all the intervention OHS units assigned to receive training in the guideline-orientated biopsychosocial management of LBP were simultaneously given a four-day initial training course and a three-day booster course at a conference venue near the city of Oulu. The first part of the training took place 21–24 September 2017 and the second part 3–5 June 2018. A senior pain psychologist and a physiotherapist with extensive experience in the biopsychosocial management of LBP delivered the training, assisted by other members of the research team. The training consisted of lectures on the theoretical basis of the biopsychosocial approach to LBP management, pain education, psychological risk factors, physical factors and behavioral responses to pain, interview and assessment, communication skills and individualized management of LBP. Demonstrations using real patients were followed by practice of clinical reasoning skills and discussions, and clinical case problem-solving. Role plays were also used to enhance learning. The use of stratification questionnaires (SBT and ÖMPSQ-short) was also practiced. The HCP who participated in the workshops were given access to an online platform with additional resources (research articles, videos etc.) and to a discussion forum. They were also able to consult the research team.

The participating physiotherapists and physicians were advised to share the information in their workplace and were provided with a printed educational package for this purpose. To support this, after the initial workshop, a member of the research team visited all the active units, with the aim of further introducing the research project to the whole workplace beyond those who participated in the training. The project was described and a short introduction to the principles of the biopsychosocial approach to the management of LBP was given. The procedure of data collection was described, and related unit-specific practical instructions were given.

The intervention units were advised to use SBT (ÖMPSQ-short only for physiotherapists) during the appointments to facilitate the individualization of care plans. The patients who consented to the trial received a patient education booklet called *Understanding Low Back Pain* [static-content.springer.com/esm/art%3A10.1186%2Fs12913-018-3526-7/MediaObjects/12913_2018_3526_MOESM3_ESM.pdf], which has been translated into Finnish and undergone preliminary evaluation in Finnish primary healthcare (21). Based on risk classification using SBT, low-risk patients were presumed to receive education on the biopsychosocial nature of pain, advice to stay active and advice on pain medication (if needed). Moderate-risk patients were supposed to receive a treatment protocol similar to low-risk patients and in addition, active physiotherapy including evaluation and guidance of the patients’ pain-related fears, functional limitations and lifestyle behaviors, such as regular physical activity, sleep, etc. High-risk patients were supposed to receive a treatment protocol similar to moderate-risk patients, but with an emphasis on exploring and integrating the management of psychosocial factors by a physiotherapist who participated in the full 7-day training. Patients could be referred to an occupational health psychologist if needed (22). The control units provided treatment as usual.

### Patient-reported outcome measures

The primary outcome measure was patient-reported back-related disability score based on the Oswestry Disability Index (ODI; 23) over 12 months. Secondary patient-reported outcome measures (PROM) – evaluated at baseline, 3 months and 12 months – included PROMIS-PF20 (PROM information system-physical function-20; 24, 25), back and leg pain intensity using a 0–10 numerical rating scale (NRS), health-related quality of life (Qol) using the EuroQol (EQ)-5D-3L (26), self-rated health (EQ-5D VAS, 0–100 scale), and work ability (0–10 NRS; 27). The Back Belief Questionnaire (BBQ; 28) was evaluated only at baseline and 3 months, whereas the Roland-Morris Disability Questionnaire (RMDQ-24; 29), the SBT (30, 31), ÖMPSQ-short (17, 32), fear of physical activity or work (Fear-Avoidance Beliefs Questionnaire, FABQ-pa and FABQ-work; 33), and self-efficacy (Pain Self-Efficacy Questionnaire, PSEQ; 34) were evaluated at baseline and 12 months. In addition, we asked the patients at all timepoints to report the use of any pain medication used at least three days per week (separate reporting for paracetamol, non-steroidal anti-inflammatory drugs, mild opioids and strong opioids). Finally, we used custom-made questions to evaluate patient satisfaction with information related to pain explanation, self-efficacy, HCP skills, and being heard and understood in terms of symptoms (0–10 NRS for all items) at all timepoints.

### Sample size

Power calculations for the trial were performed according to the primary outcome measure at 12 months. A total of 162 patients from 27 clusters (units), with an average of 6 participants (patients) each, and a conservative estimate for the intraclass coefficient of 0.05, has 80% power to detect at least a 4-point difference in ODI between the groups at alpha=0.05, assuming a standard deviation in the ODI of 8 out of 50 points (35). This equates to a 20% difference in groups at one year if the mean ODI in the control group is projected to be 20 points (Stata version 16, StataCorp LLC, College Station TX, USA). Towards the end of the recruitment, we used the accumulated data to estimate the true intraclass coefficient in our data (36) which was 0.011, suggesting that a sample size of 135 with an average of five participants per unit would have been sufficient for the assumed effect size. With an expected drop-out rate of 30%, the adjusted sample-size requirement was 192 patients.

### Randomization

The units that agreed to participate were randomized into the intervention or control groups using a random number generator, as performed by a statistician who was not aware of the characteristics of the units. Randomization was stratified by the service provider (public companies together) in an attempt to minimize selection bias. Not being involved in patient recruitment or data analysis, the last author notified the OHS providers of their allocation.

### Statistical methods

Baseline characteristics at the unit and patient levels were summarized using means and standard deviations (SD), medians and interquartile ranges (IQR), or percentages (%) and frequencies (*n*). The effects of the intervention on the primary and secondary outcomes at the patient level were estimated using 3-level linear or generalized linear mixed models with random effects for unit and time to allow for intraclass correlation at the unit and patient level, incorporating terms for intervention group, time and intervention by time interaction. The models were adjusted for delay in all variables, which showed between-group imbalance at baseline. The primary analysis used a full intention-to-treat (ITT) approach, using all available data at baseline, 3 and 12 months. As linear mixed models are a likelihood-based estimation procedure, and thus produce non-biased estimates under the assumption of data missing at random, the likely values for the missing data were estimated on the basis of the observed data. Estimates were reported with accompanying 95% confidence intervals (CI) and associated P-values, using bootstrapped standard errors to account for departures from normality. SPSS Statistics (version 26, IBM Corp, Armonk, NY, USA) and Stata were used for the statistical analyses. A Stata syntax demonstrating the primary analysis for ODI is presented in the supplementary material (www.sjweh.fi/article/3959) table S1.

## Results

### Baseline data

Figure 1 is a flowchart illustrating the progression of the study. Ten OHS providers were originally contacted, of which four declined to participate. The six participating OHS providers consented to assign 27 units in total to the trial. Two units allocated to the intervention group declined placement because they had no HCP who could participate in the training. These two units, however, agreed to participate in the control group. Finally, 12 units participated in training and 15 in the control group. Of these, 5 control units did not recruit any patients (figure 1).

Patients were recruited between 25 September 2017 and 29 November 2018. The median duration from consent to the baseline response was 24 (range 5–291) days in the intervention group and 17 (range 3–115) days in the control group. The final study population comprised 315 participants (42.5% male, mean age 45 years), with 234 and 81 patients in the intervention and control groups, respectively, at baseline. At one year, the figures were 137 and 47 patients, respectively. There were no statistically significant differences between the groups at baseline in terms of demographic characteristics, general health or work-related factors (table 1). However, 34.6% of the intervention group and 17.3% of the control group reported that their pain had lasted for >12 months (P=0.017). Pain-related fear of physical activity was lower in the intervention than control group (P=0.012; table 2).

**Table 1:** Baseline demographic, general health-related and work-related characteristics of participants. [DEPS= Depression Scale; EQ-5D, EQ-5D-3L score; LBP=low back pain].

Characteristics	All (N=315)	Between-group comparison		

		Intervention (N=234)	Control (N=81)	P-value
Demographic features				
Age ^a^ (years)	44.9 (9.9)	44.6 (9.4)	45.9 (11.2)	0.341
Female ^b^	57.5 (181)	54.7 (128)	65.4 (53)	0.092
Physically inactive ^b^	9.8 (31)	9.8 (23)	9.9 (8)	0.990
Body mass index ^a^(kg/m^2^)	27.6 (5.1)	27.7 (5.3)	27.3 (4.3)	0.527
Smoking ^b^	15.2 (48)	15.0 (35)	16.0 (13)	0.814
General health				
DEPS score ^c^	4 (2–9)	4 (2–9)	4 (2–9)	0.849
Self-rated health ^c^ (0–100)	75 (65–85)	75 (65–85)	80 (65–85)	0.327
EQ-5D ^c^ (0–1)	0.76 (0.69–0.80)	0.76 (0.69–0.80)	0.76 (0.69–0.80)	0.566
Work-related features				
Actively working ^b^	94.6 (298)	94.4 (221)	95.1 (77)	0.832
Work ability ^c^(0–10)	8 (6–9)	8 (6–9)	8 (6–8)	0.542
Sick leave due to LBP during last 3 months ^b^	51.7 (163)	49.1 (115)	59.3 (48)	0.116
Sick leave days during last 3 months ^c^	10 (4–28)	8 (4–29)	14 (4–23)	0.572
Partial sick leave due to LBP during last 3 months ^b^	14.6 (46)	14.1 (33)	16.0 (13)	0.669
Partial sick leave days during last 3 months ^c^	15 (7–60)	14 (7–60)	15 (7–38)	0.969

a Mean (standard deviation), P-value for between-group difference from independent-samples T test.

b Percentage (frequency), P-value for between-group difference from Chi square test.

c Median (interquartile range), P-value for between-group difference from Mann-Whitney U test.

**Table 2:** Baseline low back pain (LBP)-related characteristics of participants. [FABQ=Fear-Avoidance Beliefs Questionnaire; NRS=numerical rating scale; NSAID=non-steroidal anti-inflammatory drug; ODI=Oswestry Disability Index; PROMIS PF-20=patient-reported outcomes measurement information system, 20-item physical functioning short form; RMDQ=Roland Morris Disability Questionnaire; ÖMPSQ-short=short version of Örebro Musculoskeletal Pain Screening Questionnaire] **Bold denotes statistical significance.**

Characteristics	All (N=315)	Between-group comparison		

		Intervention (N=234)	Control (N=81)	P-value
Screening criteria				
Back pain intensity during past week ^a^ (NRS, 0–10)	5 (3–6)	5 (3–6)	4 (2–7)	0.832
Leg pain intensity during past week ^a^ (NRS, 0–10)	2 (0–5)	3 (0–5)	2 (0–5)	0.618
Duration of pain				
<2 weeks ^b^	13.3 (42)	11.1 (26)	19.8 (16)	
2–11 weeks ^b^	34.0 (107)	32.9 (77)	37.0 (30)	
3–12 months ^b^	22.5 (71)	21.4 (50)	25.9 (21)	
>12 months ^b^	30.2 (95)	34.6 (81)	17.3 (14)	**0.017**
LBP daily during past 3 months ^b^	54.9 (173)	55.1 (129)	54.3 (44)	0.900
Pain medication use ≥3 days during past week ^b^	37.8 (119)	35.5 (83)	44.4 (36)	0.151
Prescription for pain medication ^b^	88.9 (280)	88.5 (207)	90.1 (73)	0.682
Paracetamol ^b^	33.0 (104)	30.8 (72)	39.5 (32)	0.150
NSAID ^b^	72.4 (228)	73.1 (171)	70.4 (57)	0.639
Mild opioid ^b^	33.3 (105)	34.2 (80)	30.9 (25)	0.584
Strong opioid ^b^	1.3 (4)	0.4 (1)	3.7 (3)	0.054*
Other medication ^b^	23.8 (75)	21.8 (51)	29.6 (24)	0.154
Start Back Tool sum (risk) score ^a^	4 (2–6)	4 (2–6)	4 (3–6)	0.480
Low ^b^	47.3 (149)	47.4 (111)	46.9 (38)	
Medium ^b^	39.7 (125)	40.6 (95)	37.0 (30)	
High ^b^	13.0 (41)	12.0 (28)	16.0 (13)	0.617
ÖMPSQ-short score (risk) ^a^	38 (26–50)	38 (26–49)	41 (29–51)	0.276
Low ^b^	53.0 (167)	55.1 (129)	46.9 (38)	
Medium ^b^	21.6 (68)	20.1 (47)	25.9 (21)	
High ^b^	25.4 (80)	24.8 (58)	27.2 (22)	0.396
Pain-related fear (FABQ) – Work ^a^	12 (4–21)	11 (4–19)	13 (5–24)	0.097
Pain related fear (FABQ) – Physical activity ^a^	12 (8–16)	11 (8–14)	14 (9–17)	**0.012**
Back-pain Beliefs Questionnaire (BBQ) ^a^	31 (26–35)	31 (25–35)	31 (26–36)	0.520
Pain Self-Efficacy-beliefs Questionnaire (PSEQ) ^a^	47 (37–54)	47 (37–55)	45 (38–53)	0.411
Disability-related outcomes				
Physical impairment (RMDQ) ^a^	4 (2–8)	5 (2–8)	4 (2–9)	0.738
Physical functioning (PROMIS PF-20 T-score) ^a^	45 (42–49)	45 (42–49)	45 (41–49)	0.282
Disability (ODI, 0–100) ^a^	20 (12–28)	20 (12–28)	20 (12–29)	0.877

a Median (interquartile range), P-value for between-group difference from Mann-Whitney U test.

b Percentage (frequency), P-value for between-group difference from Chi square test (*Fisher’s exact test due to small groups).

### Patient-reported outcome measures

We observed no significant difference between the groups’ ODI, the primary outcome at 3 and 12 months [adjusted mean difference in scores +2.3 index point of the ODI score; 95% confidence interval (CI) -1.0–5.7; P=0.175 at 12 months; table 3]. There were no consistent or clinically important differences between the groups’ secondary outcome measures, except in self-rated health at 12 months (P=0.032; higher among the controls) and SBT risk-group distribution at 12 months (P=0.028; lower prevalence of high risk among the controls) (tables 3 and 4).

**Table 3:** Main outcomes and treatment effects across follow-up. [BBQ=Back Pain Beliefs Questionnaire; FABQ=Fear Avoidance Beliefs Questionnaire; EQ-5D=EQ-5D-3L score; LBP=low-back pain; NRS=numerical rating scale; ODI=Oswestry Disability Index; PROMIS PF-20=patient-reported outcomes measurement information system, 20-item physical functioning short form; PSEQ=Pain Self-Efficacy Beliefs Questionnaire; RMDQ=Roland Morris Disability Questionnaire; ÖMPSQ-short=Örebro Musculoskeletal Pain Screening Questionnaire; SD=standard deviation] **Bold denotes statistical significance**

Outcome and timepoint	Mean (SD) among groups		Between-group comparison	
	
	Intervention	Control	Adjusted mean difference ^a^ (95% CI)	P-value
LBP intensity (NRS, 0–10)				
3 months	3.9 (2.3)	3.9 (2.2)	-0.2 (-0.9–0.6)	0.651
12 months	3.8 (2.4)	3.5 (2.5)	0.2 (-0.6–0.9)	0.706
Leg pain intensity (NRS, 0–10)				
3 months	2.6 (2.7)	2.4 (2.7)	-0.1 (-0.8–0.6)	0.799
12 months	2.4 (2.4)	1.9 (2.5)	0.0 (-0.7–0.8)	0.891
ODI				
3 months	18.8 (13.2)	17.7 (10.2)	-0.3 (-3.0–2.5)	0.856
12 months	17.9 (13.1)	14.6 (10.0)	2.3 (-1.0–5.7)	0.175
PROMIS T-score				
3 months	47.0 (7.4)	46.9 (5.8)	0.7 (-0.9–2.2)	0.897
12 months	47.6 (7.2)	49.1 (6.7)	-1.0 (-2.7–0.8)	0.269
EQ-5D				
3 months	0.75 (0.18)	0.74 (0.13)	0.02 (-0.02–0.07)	0.280
12 months	0.74 (0.22)	0.78 (0.21)	-0.02 (-0.09–0.04)	0.489
Self-rated health (NRS, 0–100)				
3 months	73.0 (18.2)	76.6 (13.6)	-2.2 (-6.1–1.7)	0.276
12 months	**74.2 (17.5)**	**79.9 (15.4)**	**-4.6 (-8.9– -0.4)**	**0.032**
Work ability (NRS, 0–100)				
3 months	7.3 (2.0)	7.5 (1.4)	-0.0 (-0.4–0.4)	0.975
12 months	7.6 (2.0)	8.1 (1.7)	-0.4 (-0.8–0.0)	0.052
ÖMPSQ-short				
12 months	33.0 (17.2)	29.5 (15.4)	3.5 (-1.2–8.2)	0.140
RMDQ				
12 months	4.5 (3.8)	3.7 (3.0)	0.9 (-0.4–2.1)	0.175
FABQ -Work				
12 months	11.3 (10.7)	9.5 (9.0)	1.0 (-1.5–3.5)	0.434
FABQ -Physical activity				
12 months	9.2 (5.3)	9.0 (6.0)	0.2 (-1.4–1.8)	0.810
PSEQ				
12 months	49.0 (10.4)	50.4 (9.6)	-1.8 (-4.6–1.1)	0.223
BBQ				
3 months	30.0 (6.3)	29.6 (6.0)	0.2 (-1.5–1.9)	0.834

a Intervention vs control. Full intention-to-treat analysis: Linear mixed model, including those with no follow?up measures, incorporating baseline measure as dependent variable, and baseline response delay, duration of pain and FABQ-pa as covariates.

**Table 4:** Secondary outcomes and treatment effects across follow-up. [HCP=healthcare professional; LBP=low back pain; NSAID=non-steroidal anti-inflammatory drug; SBT=Start back tool] **Bold denotes statistical significance**

Outcome	3 months				12 months			P-value
	
	Intervention	Control	Adjusted contrast between groups ^a^	P-value	Intervention	Control	Adjusted contrast between groups^1^	
LBP daily ^b^	42.3 (58)	36.2 (17)	-0.4 (-1.5–0.8)	0.525	33.6 (44)	31.9 (15)	-0.1 (-1.3–1.0)	0.815
SBT category (risk)								
Low ^b^					**68.7 (90)**	**83.0 (39)**		
Medium ^b^					**21.4 (28)**	**12.8 (6)**		
High ^a^					**9.9 (13)**	**4.3 (2)**	**1.3 (0.1–2.4)**	**0.028**
Örebro category (risk)								
Low ^b^					65.6 (86)	72.3 (34)		
Medium ^b^					14.5 (19)	14.9 (7)		
High ^b^					19.8 (26)	12.8 (6)	0.6 (-0.5–1.7)	0.310
Medication for pain on ≥3 days/week ^b^	28.5 (39)	27.7 (13)	-0.0 (-1.3–1.2)	0.936	19.1 (25)	23.4 (11)	-0.5 (-1.7–0.8)	0.479
Prescription for pain medication ^b^	48.2 (66)	42.6 (20)	0.4 (-0.7–1.5)	0.476	71.0 (93)	66.0 (31)	0.2 (-0.9–1.4)	0.676
Paracetamol ^b^	12.4 (17)	21.3 (10)	-0.9 (-2.1–0.3)	0.127	28.2 (37)	23.4 (11)	0.5 (-0.6–1.5)	0.392
NSAID ^b^	29.9 (41)	25.5 (12)	0.4 (-0.7–1.5)	0.486	52.7 (69)	53.2 (25)	-0.1 (-1.1–0.9)	0.803
Mild opioid ^b^	15.3 (21)	12.8 (6)	0.4 (-1.3–2.1)	0.657	26.7 (35)	17.0 (8)	1.0 (-0.5–2.5)	0.204
Strong opioid ^b^	0 (0)	0 (0)	n/a	n/a	0.8 (1)	2.1 (1)	-1.2 (-4.3–1.9)	0.445
Other medication ^b^	16.1 (22)	19.1 (9)	-0.2 (-1.4–1.1)	0.812	21.4 (28)	23.4 (11)	-0.1 (-1.3–1.1)	0.882
Patient satisfaction with								
Explanation given for pain ^c^	7 (4–9)	8 (5–9)	0.7 (-0.2–1.5)	0.114	8 (3–9)	8 (5-10)	0.1 (-0.7–0.9)	0.826
Own means of controlling pain ^c^	8 (6–9)	7 (5–8)	0.4 (-0.2–1.1)	0.187	8 (5–9)	8 (7-9)	-0.5 (-1.2–0.1)	0.106
Confidence in HCP’s skills ^c^	**8 (7–10)**	**8 (6–9)**	**0.9 (0.0–1.7)**	**0.043**	8 (6–9)	8 (6-10)	0.7 (-0.2–1.6)	0.124
Being heard and understood ^c^	8 (6–9)	8 (6–9)	0.9 (-0.2–1.9)	0.100	8 (5–9)	8 (6-9)	0.4 (-0.5–1.3)	0.363

a Full intention-to-treat analysis: Linear or generalized linear mixed models as specified in the footnote, including those with no follow?up measures, incorporating baseline measure as dependent variable, and baseline response delay, duration of pain and FABQ-pa as covariates.

b Values are percentages with frequencies. Between-group difference analyzed using generalized linear (binary logistic or ordered logistic) mixed model.

c Values are medians with interquartile ranges. Between-group difference analyzed using linear mixed model with bootstrapped standard errors.

We performed additional sensitivity analyses for the main outcomes (ie, ODI, back and leg pain intensity, self-rated health and work ability) by including only those with symptom duration of >2 weeks but <12 months (N=178; sensitivity analysis 1), patients belonging to the high-risk group based on SBT (N=41; sensitivity analysis 2), or patients belonging to the SBT low-risk group (N=149; sensitivity analysis 3), and compared these subgroups to the respective control groups. The sensitivity analyses showed similar results to those of the main analysis (supplementary tables S2–4).

## Discussion

We observed no clinically relevant differences between the patient-reported outcome measures of the patients recruited by the HCP trained in biopsychosocial management of LBP and those of the patients recruited through usual OHS over one-year follow-up. Somewhat unexpectedly, the individuals in the control group reported higher self-rated health and, in the intervention group, a higher proportion of individuals were allocated to the high-risk SBT group at one-year follow-up. This may be due to several underlying factors. Firstly, the intervention group included a significantly higher percentage of individuals with pain duration of >12 months at baseline (35% versus 17%). Although pain duration was used as a covariate in all subsequent treatment effect analyses, we cannot rule out residual confounding. Secondly, it may be that the offered training encouraged professionals in the intervention units to recruit patients with more difficult symptoms overall. In contrast, professionals may have overlooked patients with demanding symptoms in the control units or these patients may have had a higher tendency to decline to participate. Possible selection biases may also relate to the study design of a cluster randomized trial: there was no subsequent randomization of patients; they were invited to participate within the randomized units. Finally, it should be acknowledged that professional competency and treatment fidelity were not assessed. In the intervention units, an HCP who was not trained by the research team may have first contacted the patient. Such HCP may have assessed psychosocial factors using SBT and given the patient the education booklet as recommended. However, the HCP who did not participate in the training may not have been able to explain pain properly and given unclear or even contradictory messages, ie, ‘mixed messages’. A previous randomized Finnish study found that a ‘Back Book’ information booklet combined with an occupational nurse appointment was no more effective than the booklet alone among patients with mild LBP symptoms, and this discrepancy could only be explained by unclear messages from the nurse (18).

The longer pain duration in the intervention group may have resulted in poorer recovery, as observed earlier among patients with LBP (37). Therefore, patients in the intervention group may have been more demanding at the start of the study, although their pain symptoms and SBT and ÖMPSQ-short risk-group distribution were similar. Moreover, there was on average a 1-week longer delay in baseline responses among the patients recruited into the training versus control group, which may have attenuated ‘true’ baseline symptom levels in the intervention group, considering the normal clinical course of LBP (38). On the other hand, the imbalance between the severity of the pain among the recruited patients in the intervention and control groups may be considered a positive phenomenon, as the physiotherapists in the intervention units seem to have been better prepared to address severe pain patients and invite the ‘more challenging’ patients to participate.

The use of relatively brief training of HCP without mentoring and long-term support means that the expectations of change in clinical practice, and especially in the patient outcomes, are limited. Previous studies have observed that often the beliefs and attitudes of the HCP change, but no change in practice and patient outcomes is achieved (39, 40). On the other hand, the training might have led to the desired change in biopsychosocial pain-management but not improved patient outcomes. Berube et al (9) reported that studies with positive patient outcomes tend to use face-to-face workshops of longer duration and include case studies and practical tools, allowing the practice of the new skills in clinical work and feedback from trainers to the participants. More intensive training and tutoring might have helped improve the transfer of the acquired knowledge into practice. Future interventions should consider supporting individual learning and problems arising during the course of learning.

It has also been suggested that changing individuals’ beliefs and competences is not enough, and successful implementation of new knowledge requires complex changes in beliefs, attitudes and clinical routines at the individual, group and organizational levels (41, 42). More research is needed to explain the ways in which implementation design should be changed to overcome clinician and organizational barriers to improve care, and in addition, to address the facilitators of practices in implementing and sustaining the change. Analysis of the facilitators and barriers will be evaluated qualitatively in order to explain what factors have influenced the implementation outcomes.

The possible effectiveness of the intervention in reducing sick leaves, imaging and visits to HCP will be evaluated later in separate analyses based on the registry data on the patients who gave consent to the use of their healthcare records. The registry-based data may be valuable for comparing the training and control groups, as the number of patients who consented to the use of their records was higher than the number of patients who responded to the baseline questionnaire, and there were no delays in the baseline responses in that data set. Relatively simple workplace educational LBP interventions have had a positive effect on work disability in earlier studies (19, 43).

We acknowledge several limitations in the current study. First, there is a strong likelihood of recruitment and selection biases. Professionals in both groups were asked to identify and recruit patients but did not keep a record of who was invited, ie, who refused to participate. Thus, a number of factors may have thwarted the ability of the trial to include similar participants in each group, with respect to their background or baseline characteristics. This is likely reflected in the higher percentage of chronic LBP cases among the patients recruited into the training group. Second, in our trial randomized units, the OHS providers asked representatives to participate in the training, and finally the representatives were advised to disseminate the information and knowledge that they had acquired during the training. This may have resulted in weaker training intervention for most of the HCP or contamination between professionals in the control versus intervention groups within the same organization. Third, there seems to have been an uneven distribution of units between the intervention and control groups. Fourth, a total of five units, all in the control group, did not recruit any patients for the study. The intervention group contained a higher number of patients than the control group, following an approximate ratio of 3:1 at baseline. Fifth, we had a relatively high dropout rate although it appeared to be similar in both groups. Furthermore, dropout during follow-up was taken into account by the statistical approach (full ITT) in the mixed model procedures. Importantly, we adjusted for any observed imbalance in baseline characteristics between the intervention and control groups at baseline in the models. Finally, we did not evaluate the biopsycho­social knowledge of HCP before the training. Thus, we are not able to document to what extent training improved their skills and knowledge.

This study also had several strengths. Few exclusion criteria can enhance generalizability in OHS. Moreover, the extensiveness of the training resembled usual training courses and was feasible. Another strength of the current study is that we included validated patient-related outcome measures for the patient sample. For example, RMDQ and ODI have good construct validity and reliability, and responsiveness over short intervals (44). The planning of this research intervention took into account the multidimensional nature of LBP and the study was designed to enhance a new approach to pain management in the OHS setting. Assessment included physical (disability), psychological (fear of physical activity, fear-avoidance, pain catastrophizing, pain self-efficacy, depression), social (work absenteeism), and health-related quality of life measures, as recommended (45).

The value of this study stems from the fact that, for the first time, we brought to Finnish OHS a multi­dimensional and -professional training intervention, in which physicians and physiotherapists were trained together for LBP patients. OHS provide an excellent context for actions at an early stage: identifying individuals at increased risk of developing prolonged pain and work disability and targeting early-stage interventions. This study provides information for both primary health care interventions and the development of HCP training within health care services in general.

In conclusion, this cluster-randomized controlled trial did not reveal reductions in LBP-related symptoms during a one-year follow-up among patients recruited by professionals trained in the guideline-oriented biopsychosocial management of LBP or among patients recruited through usual OHS. More research is required on the specific targets of the training in the clinical practice as well as the content and length of the biopsychosocial training intervention to improve patient-related outcomes. In addition, organizational level aspects should be evaluated when implementing evidence-based practice in OHS.

## Supplementary material

Supplementary material

## Trial registration

The trial is registered as ISRCTN11875357.
