# Supplementary material for: Effectiveness of training in guideline-oriented biopsychosocial management of low-back pain in occupational health services – a cluster randomized controlled trial
Source: Scand J Work Environ Health. 2021 Jun 29;47(5):367–76. doi: 10.5271/sjweh.3959 (PMC8259706; doi:10.5271/sjweh.3959)
Supplement: Supplementary material [file SJWEH-47-367-S001.pdf]

Effectiveness of training in guideline-oriented biopsychosocial management of low-back pain in occupational health services – a cluster randomized controlled trial[1]

by Katja Ryynänen, MD, PhD,[2] Petteri Oura, MD, PhD, Anna-Sofia Simula, MD, Riikka Holopainen, MSc, Maija Paukkunen, MSc, Mikko Lausmaa, BSc, Jouko Remes, MSc, Neill Booth MD, PhD, Antti Malmivaara MD, PhD, Jaro Karppinen, MD, PhD

1. *Supplementary material*
2. *Correspondence to: Katja Ryynänen, Medical Research Center Oulu, Oulu University Hospital and University of Oulu, Oulu, Finland. [E-mail: katja.ryynanen@oulu.fi]*

Table S1. A Stata syntax demonstrating the primary analysis of ODI.

Syntax	bootstrap, reps(100) seed(7777): xtmixed odi i.intervention##i.timepoint delay i.paindbaseline fabq_pa_baseline || Unit: || ID:<br>contrast i.intervention@timepoint, effects
Variables	odi = Oswestry Disability Index score<br>intervention = 0 (control group) / 1 (intervention group)<br>timepoint = 1 (baseline) / 2 (3 months) / 3 (12 months)<br>delay = baseline-response delay in days<br>paindbaseline = pain duration at baseline: 1 (<2 weeks) / 2 (2–11 weeks) / 3 (3–12 months) / 4 (>12 months)<br>fabq_pa_baseline = Fear-Avoidance Beliefs Questionnaire, Physical activity score at baseline<br>Unit = occupational healthcare unit<br>ID = Subject identification number

Table S2. Outcomes and treatment effects in sensitivity analysis 1: Individuals with low back pain duration >2 weeks and <12 months (n=178; intervention n=127, control n=51).

Outcome and timepoint	Between-group comparison	
	Adjusted mean difference (95% CI)	P value
LBP intensity (NRS, 0–10)		
3 months	-0.2 (-1.2—0.9)	0.750
12 months	-0.7 (-2.0—0.5)	0.253
Leg pain intensity (NRS, 0–10)		
3 months	0.0 (-1.1—1.1)	0.989
12 months	-0.2 (-1.3—0.9)	0.707
ODI		
3 months	-1.5 (-5.8—2.8)	0.507
12 months	0.0 (-4.9—4.9)	0.996
Self-rated health (NRS, 0–100)		
3 months	-1.3 (-6.9—4.3)	0.652
12 months	0.7 (-6.4—7.7)	0.856
Work ability (NRS, 0–100)		
3 months	0.3 (-0.2—0.8)	0.296
12 months	-0.1 (-0.6—0.5)	0.810

LBP, Low back pain; NRS, Numerical Rating Scale; ODI, Oswestry Disability Index

Table S3. Outcomes and treatment effects in sensitivity analysis 2: High risk individuals according to Start (n=41; intervention n=28, control n=13).

Outcome and timepoint	Between-group comparison	
	Adjusted mean difference (95% CI)	P value
LBP intensity (NRS, 0–10)		
3 months	0.6 (-1.1—2.2)	0.486
12 months	1.0 (-1.5—3.4)	0.450
Leg pain intensity (NRS, 0–10)		
3 months	-1.8 (-3.0—0.7)	0.223
12 months	-0.1 (-3.2—3.0)	0.947
ODI		
3 months	1.9 (-5.6—9.5)	0.616
12 months	4.3 (-8.4—17.0)	0.507
Self-rated health (NRS, 0–100)		
3 months	-9.5 (-20.5—1.5)	0.089
12 months	-11.1 (-24.3—2.1)	0.099
Work ability (NRS, 0–100)		
3 months	-0.3 (-1.4—0.8)	0.581
12 months	1.1 (-0.7—2.9)	0.244

LBP, Low back pain; NRS, Numerical Rating Scale; ODI, Oswestry Disability Index

Table S4. Outcomes and treatment effects in sensitivity analysis 3: Low-risk individuals according to STarT Back Tool (n=149; intervention n=111, control n=38).

Outcome and timepoint	Between-group comparison	
	Adjusted mean difference (95% CI)	P value
LBP intensity (NRS, 0–10)		
3 months	-0.2 (-0.9—0.6)	0.651
12 months	0.2 (-0.6—0.9)	0.706
Leg pain intensity (NRS, 0–10)		
3 months	-0.1 (-0.8—0.6)	0.799
12 months	0.1 (-0.7—0.8)	0.891
ODI		
3 months	-0.3 (-3.0—2.5)	0.856
12 months	2.3 (-1.0—5.7)	0.175
Self-rated health (NRS, 0–100)		
3 months	-2.2 (-6.1—1.7)	0.276
12 months	-4.6 (-8.9—-0.4)	0.032
Work ability (NRS, 0–100)		
3 months	-0.0 (-0.4—0.4)	0.975
12 months	-0.4 (-0.8—0.0)	0.052

Bold denotes statistical significance. LBP, Low back pain; NRS, Numerical Rating Scale; ODI, Oswestry Disability Index
